# Affective Meaning, Concreteness, and Subjective Frequency Norms for Indonesian Words

**DOI:** 10.3389/fpsyg.2016.01907

**Published:** 2016-12-06

**Authors:** Agnes Sianipar, Pieter van Groenestijn, Ton Dijkstra

**Affiliations:** ^1^Donders Institute for Brain, Cognition and Behaviour, Radboud University NijmegenNijmegen, Netherlands; ^2^Faculty of Psychology, Universitas IndonesiaDepok, Indonesia; ^3^Research Technical Support Group, Faculty of Social Sciences, Radboud University NijmegenNijmegen, Netherlands

**Keywords:** emotion, semantic, rating study, Indonesian, cross-cultural study

## Abstract

This study investigated the lexical-semantic space organized by the semantic and affective features of Indonesian words and their relationship with gender and cultural aspects. We recruited 1,402 participants who were native speakers of Indonesian to rate affective and lexico-semantic properties of 1,490 Indonesian words. Valence, Arousal, Dominance, Predictability, Subjective Frequency, and Concreteness ratings were collected for each word from at least 52 people. We explored cultural differences between American English ANEW (affective norms for English words), Spanish ANEW, and the new Indonesian inventory [called CEFI (concreteness, emotion, and subjective frequency norms for Indonesian words)]. We found functional relationships between the affective dimensions that were similar across languages, but also cultural differences dependent on gender.

## Introduction

Language is a vehicle that we use to present or conceal our emotional states or to communicate emotional states in social interactions (Koelsch et al., 2015). As constituents of language, words do not only have abstract semantic or referential meanings (Finegan, 2008), but also convey the emotional quality of their underlying concepts/references (e.g., they also have connotative/affective meaning). A review on studies of emotion word processing (Citron, 2012) indicates that the extent to which the effects of words’ affective dimensions modulate various cognitive processes, such as learning, memory, and attention, has become an active topic of research. To understand the mechanisms underlying affective processing, there is a need for norming datasets that provide stimulus ratings on various affective and non-affective qualities of words in various target languages. These allow researchers to generate well-controlled sets of emotional and non-emotional verbal stimuli, and to test the differential effects of affective word dimensions in various cognitive tasks. Precisely these considerations underlie our efforts to establish a normative set of affective and psycholinguistic norms for Indonesian words in the present study.

The first empirical study to investigate the affective substrates of word meanings applied the so-called “semantic differential” to written language (Osgood, 1952; Osgood and Suci, 1955; Osgood et al., 1957). The study used a dimensional approach to factor emotional aspects of visual stimuli. To extract dimensions underlying the affective meanings of words, Osgood et al. (1957) collected American-English speakers’ ratings of words using 1-to-7 scales of 50 bipolar/antonym pairs (e.g., good–bad). In a factor analysis of the scales’ ratings, most of the variance was accounted for by three major semantic dimensions, namely *Evaluation* (good–bad), *Activation* (active–passive), and *Potency* (strong–weak). Subsequently, these bipolar dimensions have consistently appeared in the factorization of affective word meaning across 20 languages/cultures (Osgood, 1962; Osgood et al., 1975).

The idea of mapping affective meaning onto a multidimensional space is compelling to psychologists working on models of emotion. For example, the circumplex model of affect (Russell, 1980) proposes that emotion concepts (e.g., happy, sad) are best mapped onto a two-dimensional semantic space, involving *Pleasure* (valence quality)—whether the stimulus is pleasant (positive) or unpleasant (negative), and *Arousal*—the degree of activation (high or low). Moreover, these two affective dimensions have been considered as separate or orthogonal in the light of their low correlation (Feldman Barrett and Russell, 1998). The general presence of Valence and Arousal dimensions has been confirmed through comparative study with different cultural groups or language speakers (Russell, 1983).

The suitability of the dimensional approach in studies on emotion-laden stimuli was also supported by the Bioinformational theory of emotion (Lang, 1993, 1995). In this approach, emotions are defined as action dispositions that are contingent on two “primary motive” systems: the appetitive and aversive/defensive motivational systems. Together they represent the Valence system (Lang et al., 1997) as the primary explanatory factor for affective responses. In contrast to Feldman Barrett and Russell (1998), the Bioinformational model considers Arousal as a system that is not separate from Valence, but “representing activation (metabolic and neutral) of either the appetitive and aversive systems, or the coactivation of both systems.” (Lang et al., 1997, p. 101). Based on findings from rating studies for words (Bradley and Lang, 1999a), pictures (Lang et al., 1999), and sounds (Bradley and Lang, 1999b), it has been suggested that the affective aspect of stimuli consists of three major dimensions that account for most of the variances in the affective ratings, namely *Valence*, *Arousal*, and *Dominance*; although Dominance, which is parallel to *Potency* (Osgood et al., 1957) or the degree of feeling in-control (Bradley and Lang, 1999a), did not play a significant role in the factor analysis. However, other researchers have argued for the importance of Dominance in distinguishing emotion concepts that belong to similar ends of Valence scale (e.g., anger and fear; Demaree et al., 2005). As such, Dominance remains a crucial dimension in many affective rating studies even today.

In addition, a fourth emotional dimension, called *Unpredictability* has consistently appeared in a cross-cultural study that involved emotion words in Dutch, English, and French (Fontaine et al., 2007). Fontaine et al. (2007) investigated whether the interrelation among emotion words across the different languages would yield the traditional three-dimension space or a set of more dimensions. It turned out that speakers of Dutch, English, and French categorized emotion words better in four dimensions: Valence, Arousal, Potency, and Unpredictability. This finding suggests that Unpredictability should be included as one of the affective dimensions underlying word meaning. Moreover, Unpredictability was suggested to be parallel to Uncertainty, a dimension that has also been investigated in many appraisal theories (e.g., Smith and Ellsworth, 1985). This dimension is crucial to distinguish emotion words in terms of the extent to which an emotion reflects reactions to an unfamiliar situation or a novel stimulus, for instance, fear or anger (Fontaine et al., 2007). Unfortunately, the uniqueness of this dimension and its relation to other affective dimensions of word meaning have never been explored in previous word norming studies.

In addition to providing a normed emotion-laden word dataset for the Indonesian language, the present study also intends to explore the generalizability of findings from earlier database studies on affective dimensions. To achieve these aims, we collected the first set of concreteness, emotion, and subjective frequency norms for Indonesian words (abbreviated as CEFI). In the following sections, we explain the research issues that we subsequently explore through the CEFI database. Specifically, the present study zooms in on the relationships between affective dimensions and between affective and non-affective aspects of word meanings in Indonesian speakers, and the extent to which these affective dimensions are correlated across gender and cultures. Our focus is on comparisons between Indonesian, American English, and Spanish speakers. We first present a review of findings regarding the relationships between affective dimensions, the relationships between affective and non-affective dimensions, and the correlations of affective dimensions in Indonesian men and women. Next, we consider the correlations across cultures and genders. Finally, we discuss the implications of our findings for research on emotion-laden word processing.

### The Cross-Cultural Aspect in Valence, Arousal, and Dominance Ratings

The dimensional approach to clarify relationships between different emotions (Smith and Ellsworth, 1985) has been applied to various languages in European cultures in order to elicit ratings of the emotional content of words for various numbers of participants. Most affective rating studies assessed the valence and arousal levels of words in European languages, such as French (Monnier and Syssau, 2014, involving 469 participants; Ric et al., 2013, *N* = 328), German (Grühn and Smith, 2008, involving 24 young adults, 24 older adults, and 19 additional participants; Võ et al., 2009, *N* = 200), English (Eilola and Havelka, 2010, *N* = 304; Warriner et al., 2013, *N* = 1,827), Finnish (Eilola and Havelka, 2010, *N* = 304; Söderholm et al., 2013, *N* = 996), Dutch (Moors et al., 2013, *N* = 224), Spanish (Ferré et al., 2012, *N* = 504; Guasch et al., 2015, *N* = 826; Hinojosa et al., 2016, *N* = 660; Stadthagen-Gonzalez et al., 2016, *N* = 512), and Polish (Imbir, 2015, 2016, involving 1,670 and 400 participants; Riegel et al., 2015, *N* = 266). Some studies also utilized a selective set of emotional stimuli, such as taboo words (Janschewitz, 2008, *N* = 84) and antonym pairs (Citron et al., 2012, *N* = 82). As for now, the affective norms for English words (ANEW) dataset has been adapted to several other languages, namely European Portuguese (Soares et al., 2012, *N* = 958), Italian (Montefinese et al., 2014, *N* = 1,084), German (Schmidtke et al., 2014, *N* = 185), and Spanish (Redondo et al., 2007, *N* = 720). The ANEW dataset has also been extended from 1,034 words (Bradley and Lang, 1999a) to 2,476 words (Bradley and Lang, 2010). However, to date, no published norming study is available that comprises both affective and lexico-semantic properties for words of any non-European language.

Since 1928, Bahasa Indonesia (the Indonesian language) has been decreed as the national language of the world’s fourth most-populous nation in the world (Sneddon, 2003; Winskel and Widjaja, 2007). Based on its linguistic typology, Indonesian language is categorized as one of the Austronesian languages (Winskel and Widjaja, 2007). In terms of how individual members of a cultural group consider the relation between one’s self and others, Indonesian, seen as a cluster of members of many ethnic groups, has been classified as one of the world’s collectivistic cultures in which the distinction between self and others is blurred; whereas speakers of languages of Western nations, such as Spain and United States, are part of individualistic cultures that view the self as independent from its surrounding interpersonal context, be it one’s family or other communities (Suh et al., 1998).

Furthermore, previous studies have shown differences in how individualistic and collectivistic cultures shape the self-construal of their members and their appraisals toward emotional experiences. For example, it has been suggested that emotional experiences are *more salient* to members of individualistic cultures than members of collectivistic cultures (Kuppens et al., 2008). This cultural difference might be related to the different display rules in expressing emotions in the two culture types and the strength of the display rules in everyday life (Fischer and Manstead, 2000; Matsumoto et al., 2010). There is a common assumption in Asian cultures that inhibiting emotional expressions is necessary, especially when such expressions potentially disturb other members of the group (Matsumoto et al., 2010). Such regulation of emotional expressions is less strongly adhered to by members of individualistic cultures (Fischer and Manstead, 2000; Matsumoto et al., 2010). A study involving Indonesian Minangkabau people (men only) and Americans (men and women) showed that both cultural groups showed similar autonomic nervous system patterns and emotion-specific instructed facial configurations during the Directed Facial Action task. However, Minangkabau participants were less likely than Americans to report that they experienced the target emotions induced by the instructed facial configurations (Levenson et al., 1992).

Such cultural differences are interesting to investigate in the light of studies on cross-cultural correlational results on words’ affective dimensions between Indo-European languages. Across European cultures, thus far, Valence, Arousal, and Dominance were significantly correlated, with the highest correlational estimates for Valence dimension (Redondo et al., 2007; Soares et al., 2012; Montefinese et al., 2014). It is, nevertheless, important to find out if cross-cultural correlations between Indonesian words’ ratings and previous ratings from other western cultures also show similar patterns on these affective word dimensions.

### Relationships between Affective Dimensions

According to the original ANEW study (Bradley and Lang, 1999a), a two-dimensional space that involves Valence and Arousal dimensions shows a quadratic relationship between the two variables: words that fall in the lower and the higher ends of Valence scale have a tendency to be perceived as more arousing than neutrally valenced words. Valence also has a positive linear relationship with Dominance, suggesting that words associated with higher Valence ratings (positive valence) tend to be rated as more dominant than words associated with lower Valence ratings (Grühn and Smith, 2008; Moors et al., 2013; Warriner et al., 2013; Montefinese et al., 2014). These result patterns have been replicated in other norming studies for various European languages.

However, findings on the relationship between Arousal and Dominance ratings have been mixed; some studies observed a quadratic relationship between Arousal and Dominance (Warriner et al., 2013; Montefinese et al., 2014), other studies found a positive linear relationship (Moors et al., 2013; Imbir, 2015), while one study (Grühn and Smith, 2008) reported a weak, non-significant linear relationship. In addition, Moors et al.’s (2013) analysis on the original ANEW database showed a weak albeit significant positive linear relationship between Arousal and Dominance.

### The Relationship between Affective Dimensions and Gender

As mentioned above, many studies found high variability in the ratings of Arousal and Dominance. Aside from a possible confounding effect of cultural differences, another factor that might contribute to such variability is gender (Montefinese et al., 2014). Across cultures, personality traits can be divided in terms of their association to gender types, such as femininity and masculinity. Femininity is considered as more intuitive, emotional, and weak, whereas masculinity is associated with traits such as rational and strong (Fischer and Manstead, 2000). Such division of traits has pervasive effects in the characterization of the expected social roles for women and men across the globe. However, not all cultures exercise similar levels of adherence toward the division of gender roles. Specifically, among collectivistic cultures in Asia, South America, and Africa, gender-related social roles are more traditional than they are among individualistic cultures (Fischer and Manstead, 2000). Furthermore, it has been shown that, unlike males from individualistic cultures, males from collectivistic cultures (e.g., Asian males) learn from a young age on how not to display their emotion (e.g., Shea and Yeh, 2008).

Thus far, much less attention has been devoted to understanding the extent to which gender might influence the contribution of cultural differences in affective responses toward verbal stimuli. Studies conducted in European cultures on the relationship between gender and affective dimensions of word meaning have found significant correlations between men and women for Valence, Arousal, and Dominance. However, weak correlational results between the two gender groups have also been reported for Arousal and Dominance (Warriner et al., 2013; Montefinese et al., 2014). In contrast to Valence, Arousal, and Dominance consistently also showed weaker though significant cross-cultural correlations for men and women (Redondo et al., 2007; Soares et al., 2012). Therefore, compared to the other dimensions, Valence seems to be the most stable affective property of word meaning both across cultures and gender groups. To generalize this hypothesis to speakers of non-European languages, the current study considers correlations for each of the affective dimensions between Indonesian, Spanish, and American women and men. Possibly, the weak correlations between men and women in Arousal and Dominance dimensions are also found in the affective ratings of Indonesian words. Furthermore, the strongest correlations between the ratings of men or women from Indonesia and those from European or western cultures, such as American and Spanish cultures, are expected to arise on the Valence dimension.

### Relationships between Affective and Other Lexico-Semantic Dimensions

In the last decade, research has shown that word processing is influenced by an interaction of affective and lexico-semantic aspects of words. Lexico-semantic aspects of words concern, for instance, Concreteness and Familiarity. The construct of the subjective familiarity of words is often paralleled with that of Subjective Frequency (Brysbaert et al., 2016). In a Familiarity rating, people are asked to rate how familiar they are with a word, whereas in a Subjective Frequency rating, people are asked to rate how frequently they encountered a word (e.g., subjective frequency). The latter type of instruction has been suggested to be more straightforward and clearer to the participants (Balota et al., 2001). Therefore, in the present study we used an instruction similar to that of Balota et al. (2001) to collect ratings of Subjective Frequency.

According to previous findings, Familiarity has a linear relationship with both Valence and Arousal (Citron et al., 2012; Stadthagen-Gonzalez et al., 2016) in the sense that more familiar words tend to have higher levels of Valence but lower levels of Arousal. However, a very weak linear relationship between Arousal and Familiarity has also been reported (Guasch et al., 2015). In the current study, we expect Subjective Frequency to be linearly correlated with Valence and Arousal. With respect to the subjective rating of Concreteness, abstract words tend to be perceived as more emotionally valenced than concrete words (Kousta et al., 2011), suggesting that Valence and Concreteness might not merely have a linear relationship (Hinojosa et al., 2016), but possibly a quadratic one. To our knowledge, such a quadratic relationship between Valence and Concreteness has been reported only in one rating study on Polish words (Imbir, 2016). With respect to Arousal, we predict a linear relationship between Arousal and Concreteness: abstract words should be rated as more arousing than concrete ones (Guasch et al., 2015; Stadthagen-Gonzalez et al., 2016).

## Materials and Methods

### Participants

In total, 1,402 native Indonesian speakers (772 females, age range: 17–42 years old, mean = 20 years old) participated in the study. The participants were university students in West Java (208 people), Jakarta (690 people), North Sumatra (325 people), Central Java (115 people), South Sumatra (one person), and Banten (two people). There were also two participants living outside Indonesia. Additionally, 59 participants did not identify their academic institutions. Out of the 1,402 participants, 1,229 were recruited through research assistants and they received small monetary compensation (1.5 Euros). The rest of the participants were volunteers who came across the researcher’s blog on Internet and participated without any monetary compensation. The Ethics Committee of the Faculty of Social Sciences of Radboud University Nijmegen approved this study. A profile of our participants on each dimension is given in **Table 1**. Comparison (*t*-test) analyses showed that the age difference between female and male participants on each dimension was small but significant (all *p*s < 0.05): male participants tended to be older than female ones.

**Table 1:** Summary of gender and age distributions in each dimension.

	Women			Men		
	Number of participants	Age range	Mean age *(SD)*	Number of participants	Age range	Mean age *(SD)*
Valence	177	17–29	19.9 (1.6)	151	17–42	20.6 (3)
Arousal	205	18–30	19.7 (1.6)	192	17–36	20.9 (2.2)
Dominance	209	17–30	19.8 (1.5)	172	17–30	20.8 (2)
Predictability	167	18–23	19.5 (1.1)	149	18–31	21 (2.3)
Concreteness	193	17–24	19.5 (1.3)	169	18–31	20.9 (2.1)
Frequency	194	17–28	19.7 (1.4)	180	18–36	20.8 (2.7)

### Materials and Design

The database consisted of 1,490 Indonesian words, of which 637 words were translations of English words in ANEW (Bradley and Lang, 2010). The words were first divided into two sets of five lists (in total 10 lists) and each list consisted of 298 word items. A list of 298 words was assessed on a 1-to-9 scale that represented one of the six rating variables: Valence (negative–positive), Arousal (calm–active), Dominance (controlled–in control), Subjective Frequency (rare–frequent), Concreteness (concrete–abstract), and Predictability (unpredictable–predictable). We applied a web survey procedure using Perseus Survey Solutions software, which enabled us to randomize the order of the lists and the order of the scales with respect to particular variables. In this way, the possibility that participants, who participated more than once, received the same word list for the same scale was almost excluded.

### Procedure

By way of introduction, participants were explicitly informed about the goals of the survey, the name of the researchers, and e-mail contacts. The participants were also informed about the filling and rating instructions, the estimated time to complete the survey, and the confidentiality of personal data. The participants were explicitly informed that they were free to participate and to quit in the middle of completing a questionnaire. Finally, if they liked to participate in the survey, they were asked to click on the button “submit.” Upon clicking this button, one of the 10 lists would be randomly chosen by the software, after which one of the 6 rating variables would be randomly assigned and presented on the right side of the word list. Each participant was asked to carefully read the instruction prior to rating the words based on the presented scale.

## Results and Discussion

In this section, we first describe the statistical characteristics and reliability indexes of the rating scales. Next, we describe the functional relationships between affective dimensions within Indonesian speakers, the extent to which Indonesian affective dimensions relate to the lexical dimensions of Concreteness and Subjective Frequency, and how Valence, Arousal and Dominance dimensions differ between Indonesian males and females. Finally, we assess what the cross-cultural differences are within male and female groups. The Supplemental Material (Data Sheet 1/CEFI) includes all values for the assessments of Valence, Arousal, Dominance, Predictability, Concreteness, and Subjective Frequency. The rating instructions are provided in Data Sheet 2 (Indonesian) and Data Sheet 3 (English).

### Descriptive Statistics

**FIGURE 1:**
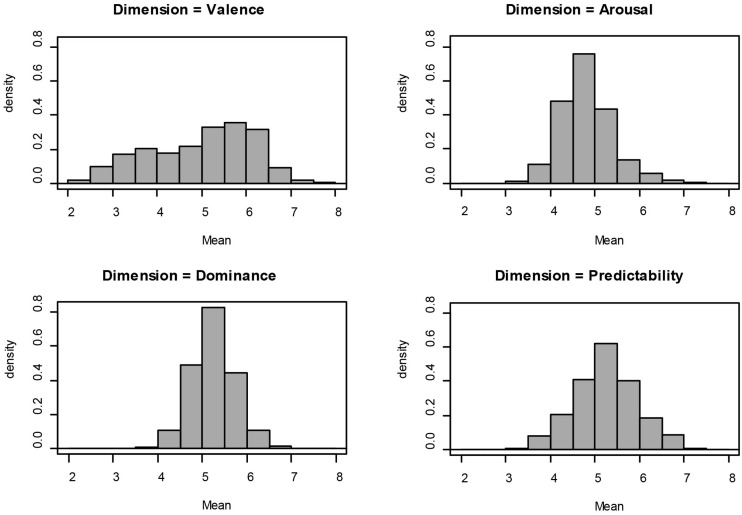
**Distribution of emotional dimension ratings**.

**FIGURE 2:**
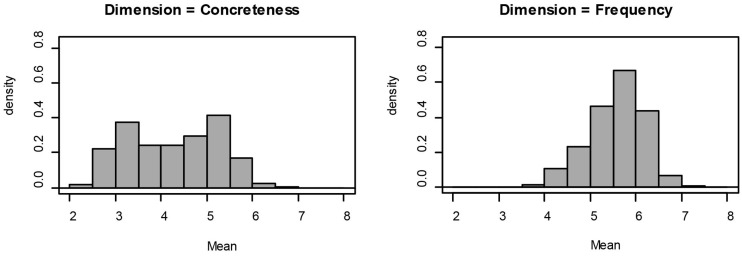
**Distribution of Concreteness and Frequency ratings**.

**FIGURE 3:**
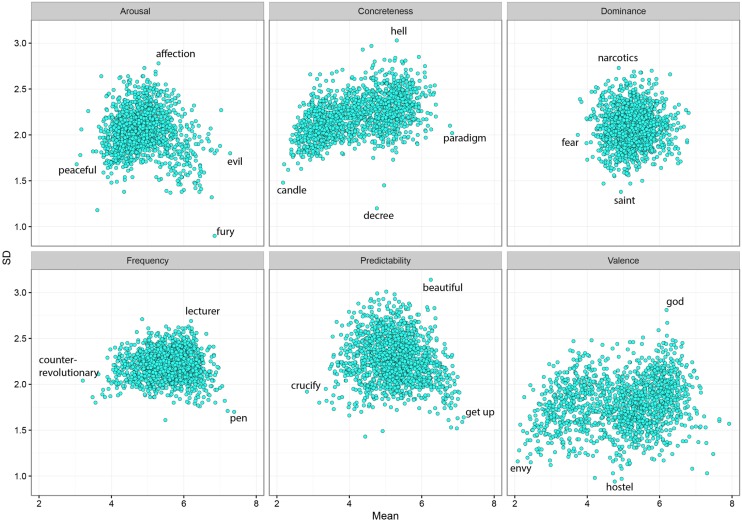
**Means of ratings plotted against the SDs for all 1,490 Indonesian words**.

Descriptive statistics of the variables are presented in **Table 2**, the density distribution for every rating variables are presented in **Figures 1** and **2**, and the scatterplots for all rating variables are presented in **Figure 3**. For interpretation of the scatterplots, we conducted regression analyses in order to explain the relationship of each rating variable and its standard deviation (SD). In addition, the magnitude of Pearson correlation coefficients will be interpreted based on Hemphill (2003).

**Table 2:** Summary of rating dimensions with means, standard deviations (SD), range, and median.

Dimension	Mean	*SD*	Range	Median
Valence	4.97	1.79	2.08–7.92	5.20
Arousal	4.86	2.04	3.04–7.28	4.79
Dominance	5.25	2.09	3.74–6.80	5.23
Predictability	5.24	2.27	2.83–7.16	5.24
Concreteness	4.27	2.21	2.31–6.84	4.27
Frequency	5.57	2.21	3.21–7.39	5.64

The Valence dimension was characterized by a negatively skewed distribution (*G*_1_ = -0.38), wherein 43.6% of the words were rated below the score of 5. The density histogram showed a tendency for a bimodal distribution with two peaks: one for the scores of 3 to 5 and another one for 5 to 6.5. The Valence scatterplot showed a weak but significant linear relationship between the mean ratings and the SDs. Words associated with higher valence ratings tended to be rated with higher SDs, such as “*god*,” *R*^2^ = 0.03, *r* = 0.16, *p* < 0.001. For the Arousal dimension, the distribution in the density histogram was positively skewed (*G*_1_ = 0.62). Here 66.6% of the words were rated below the score of 5. Meanwhile, the Arousal scatterplot showed a significant quadratic relationship between the ratings and SDs, *R*^2^ = 0.11, *r* = 0.33, *p* < 0.001. The average consensus for words associated with the lowest and highest ratings was higher (lower SD) than for words associated with ratings near the median of the scale. For Dominance, the density histogram also showed a slight tendency for a positively skewed distribution (*G*_1_ = 0.13), as only 29.4% of the words were rated below 5. The plot for Dominance ratings showed a radial pattern in which neither quadratic nor linear fits yielded any significant relationship between the rating means and their SDs, *p*s > 0.1. For Predictability, there was an approximately normal distribution of ratings (*G*_1_ = -0.01). Here 34% of the words were rated with a score below 5. The scatterplot for Predictability and its SD showed a moderately strong quadratic relationship, *R*^2^ = 0.1, *r* = 0.28, *p* < 0.001. Words associated with a predictable feeling such as “*get up*,” and words associated with an unpredictable feeling such as “*crucify*,” were associated with lower SDs than words associated with Predictability scores around the median of the scale, such as “*beautiful*.”

On the distribution of Concreteness ratings, 69% of the words were rated below the score of 5 (*G*_1_ = -0.02). Two modes of ratings, namely in the interval of 3 to 3.5 and 4.5 to 5.5, were observed. The Concreteness scatterplot shows a tendency for increasing SDs with increasing ratings of Concreteness (from concrete to abstract), as indicated by a strong linear fit, *R*^2^ = 0.2, *r* = 0.47, *p* < 0.001. Meanwhile, for Frequency ratings, 17.2% of the words were rated below the scale’s median score, suggesting that the distribution pattern was negatively skewed (*G*_1_ = -0.47). The plot for Frequency showed a weak but significant quadratic relationship between ratings and SDs, *r* = 0.2, *p* < 0.001. The lowest SDs were observed for lower and higher Frequency ratings. These findings suggest that words rated as subjectively more or less frequently used, were associated with higher agreement across speakers, compared to words whose subjective frequency was rated around the middle of the scale (e.g., score of 5).

### Reliability

To assess the reliability of our ratings, we also computed the split-half reliability for each presentation list separately. For each list, word ratings for individual raters were divided into two groups. In each group, we calculated the mean rating for each word and then correlated the means of the groups. In general, the Spearman–Brown corrected correlations ranged from *r* = 0.91 to *r* = 0.95 for Valence; *r* = 0.94 to *r* = 0.97 for Arousal, *r* = 0.88 to *r* = 0.99 for Dominance; *r* = 0.92 to *r* = 0.99 for Predictability; *r* = 0.97 to *r* = 0.99 for Subjective Frequency; and *r* = 0.88 to *r* = 0.97 for Concreteness (all *p*s < 0.001). These correlational results indicate that the ratings of each word list were highly reliable. We could not extend our reliability tests by correlating ratings in CEFI with another dataset, as there has been no other published Indonesian word rating datasets that involve any of the variables.

### Relationships between Affective Variables

**FIGURE 4:**
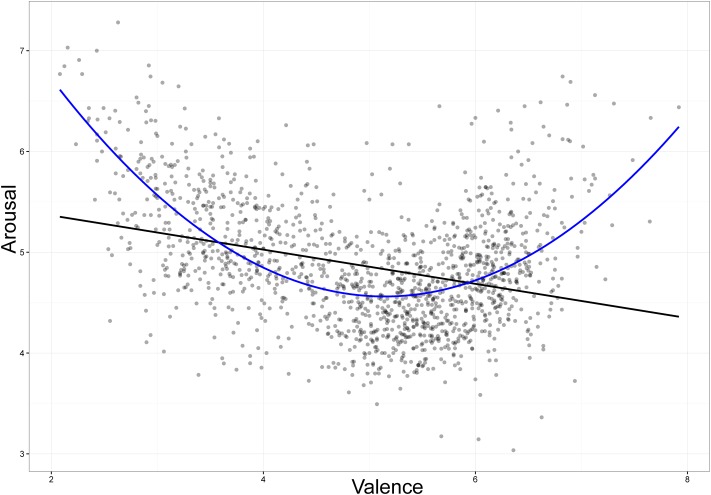
**Valence ratings plotted against Arousal ratings**.

First, we considered the relationship between Valence and Arousal through regression analyses with Valence as the independent variable and Arousal as the dependent variable. As predicted, the two variables showed the strong quadratic relationship, *r* = 0.58, *p* < 0.001. Thus, this trend accounted for 34% of the variance, while a linear regression accounted for only 10% of the variance (**Figure 4**). The pattern was confirmed in our simple regression analyses between Valence and Arousal for unpleasant words (mean = 3.61, SD = 0.57, range = 2.08–4.56), *r* = -0.42, *p* < 0.001, for neutral words (mean = 5.21, SD = 0.31, range = 4.57–5.69), *r* = -0.10, *p* < 0.05, and for pleasant words (mean = 6.20, SD = 0.36, range = 5.70–7.92), *r* = 0.32, *p* < 0.001. This distribution fits the typical U-shape found in previous studies of American English and European languages (e.g., Bradley and Lang, 1999a; Redondo et al., 2007). This indicates that Indonesian speakers also rate highly pleasant or highly unpleasant words as more arousing than neutral ones.

**FIGURE 5:**
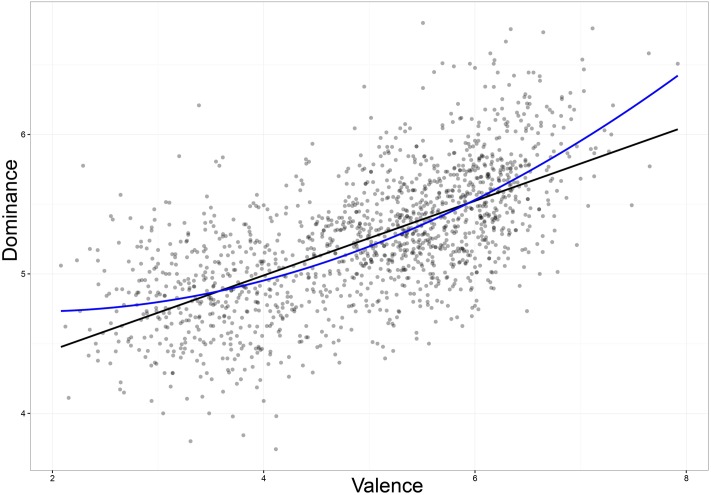
**Valence ratings plotted against Dominance ratings**.

Regression analysis on Valence and Dominance ratings resulted in a strong linear relationship between the two, *r* = 0.64, *p* < 0.001. This accounts for 41% of the variance. A quadratic regression accounted for 43% of the variance, a relatively small increase compared to the linear analysis (**Figure 5**). Our findings confirm the strong positive linear relationship between Valence and Dominance reported in previous studies (Grühn and Smith, 2008; Moors et al., 2013; Warriner et al., 2013; Montefinese et al., 2014).

The relationship between Arousal and Dominance indicated a rather weak but highly significant quadratic fit, *r* = 0.25, *p* < 0.001, accounting for only 6% of the variance. The linear relationship between Arousal and Dominance was even weaker, but significant (*p* < 0.001). Further analyses showed that a negative correlation emerged between low-dominant words (mean <5.22) and Arousal levels, *r* = -0.22, *p* < 0.0001, whereas high-dominant words (mean ≥5.22) were positively correlated with Arousal levels, *r* = 0.2, *p* < 0.0001. These patterns are in line with a previous rating study (Warriner et al., 2013) that reported a quadratic relationship between Arousal and Dominance. The direction of the relationship between Arousal and Dominance has been inconsistent across word rating studies, with most of them showing a positive linear correlation (e.g., Moors et al., 2013). However, it is worth considering that the rating distributions of these two dimensions varied quite considerably across cultures. Imbir (2015) noted that a confounding effect of cultural differences might affect how these dimensions relate to each other.

Moreover, Valence and Predictability showed a positive linear relationship, *r* = 0.34, *p* < 0.001, similar to the relationship between Dominance and Predictability, *r* = 0.31, *p* < 0.001. In contrast, Arousal and Predictability showed a moderately strong and significant negative linear relationship, *r* = -0.28, *p* < 0.001. These findings suggest that words that were perceived as positive and dominant tended to be rated as more predictable. However, the more arousing a word is, the more likely it is perceived as unpredictable. Specifically, the finding also suggests that there is an arousal bias in how Indonesian speakers perceive the relative predictability of words’ affective meaning.

### Relationships between Valence/Arousal and Lexico-Semantic Variables

**FIGURE 6:**
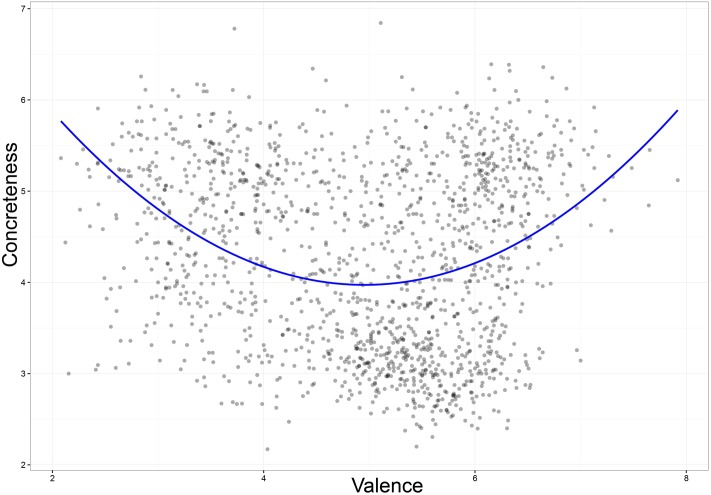
**Valence ratings plotted against Concreteness ratings**.

The relationship between Valence and Concreteness was quadratic, *r* = 0.31, *p* < 0.001, which accounted for 9.8% of the variance. The significant linear regression only accounted for 1% of the variance (**Figure 6**). This finding is in line with Imbir (2016) as well as Kousta et al. (2011), who suggest that abstract words tend to be more emotionally valenced than the concrete ones. On the other hand, a regression analysis on Arousal and Concreteness yielded a positive linear relationship, *r* = 0.36, *p* < 0.001. Thus, abstract words tend to be rated as more arousing (Guasch et al., 2015; Stadthagen-Gonzalez et al., 2016). With respect to Subjective Frequency, a regression analysis on the ratings of Valence and Subjective Frequency showed a positive linear relationship, *r* = 0.28, *p* < 0.0001; whereas neither the correlational nor the regression analyses between Arousal and Subjective Frequency were significant (*p* > 0.1). The findings suggest the presence of a positivity bias, but not an arousal bias, in the subjective frequency of word use in Indonesian speakers. A previous study also reported that less familiar words tend to fall in the negative words category (Ferré et al., 2012).

### Correlations between Indonesian Gender Groups

**FIGURE 7:**
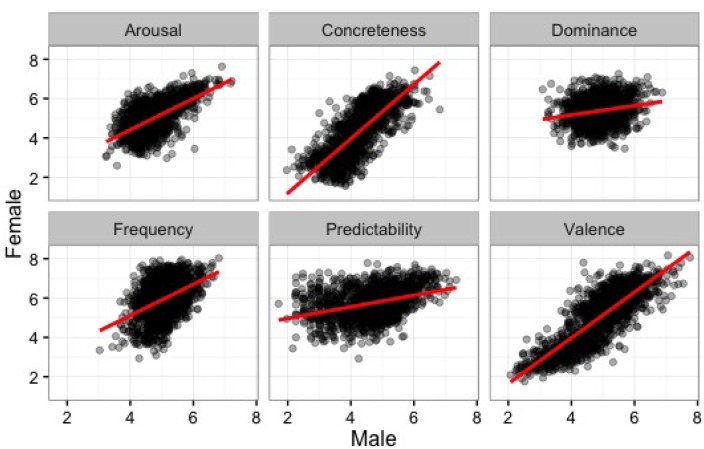
**Male ratings for each dimension plotted against female ratings**.

We also conducted correlational tests to understand the relationship between female and male ratings for each rating variable. In general, there were strong correlations in the two gender groups’ ratings for Valence, *r* = 0.84; Arousal, *r* = 0.6; Predictability, *r* = 0.36; Concreteness, *r* = 0.79; and Subjective Frequency, *r* = 0.43. The correlation was weaker, however, for Dominance, *r* = 0.2, all *p*s < 0.01 (see **Figure 7**). These findings suggest that Indonesian men and women display similar patterns in their ratings of the affective and non-affective aspects, such as Concreteness and Subjective Frequency, of Indonesian words. Additional correlational analyses on all dimensions after removing ratings from participants older than 25 years of age yielded comparable results. It is important to note that the less strong correlation between men and women in Dominance might be due to the different cultural rules in expressing feeling in-control between the two gender groups in daily life. We will discuss this possibility in the next section, where we take a look at the cross-cultural aspects for each affective dimension through simple regression analyses on the ratings of men and women in Indonesia, United States, and Spain.

### Cultural and Gender-Based Regression Analyses

#### Cultural Analyses

Several regression analyses were run to understand the correlations and similarity between cultures, by comparing ratings of Valence, Arousal, and Dominance in Indonesian speakers, American English (ANEW, 2010), and Spanish speakers (Spanish adaptation of the original ANEW) on the 637 words that are common in all three datasets. As can be seen in **Table 3**, Indonesian speakers’ affective ratings (collapsed across the two genders) were highly correlated to those of American and Spanish speakers. As in previous studies (Redondo et al., 2007; Soares et al., 2012), Valence showed the highest correlations across cultures, followed by Arousal and Dominance. More importantly, the strength of the correlational estimates were comparable to previous cultural comparisons of ANEW (Redondo et al., 2007; Soares et al., 2012; Montefinese et al., 2014).

**Table 3:** Cross-cultural correlational results (collapsing gender).

Dimension	USA	Spain
**Valence**		
Indonesia	0.85	0.84
USA		0.91
**Arousal**		
Indonesia	0.58	0.60
USA		0.76
**Dominance**		
Indonesia	0.64	0.63
USA		0.73

*All correlations are significant at α = 0.05 (two-tailed)*.

#### Cultural Analyses by Gender

**FIGURE 8:**
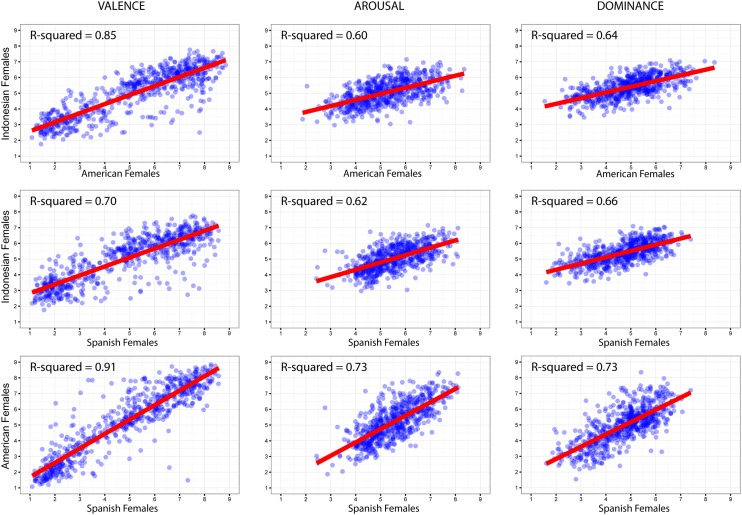
**Cross-cultural regression analyses on women’s ratings**.

Next, we consider the cross-cultural regression analyses in men and women, separately. As can be seen in **Figure 8**, the correlations across cultures in the women group were fairly high across dimensions, ranging from *r* = 0.77, *R*^2^ = 0.6, to *r* = 0.92, *R*^2^ = 0.85 (all *p*s < 0.001). As predicted, Valence still showed the highest correlations and accounted the most variances between the ratings of Indonesian and American women, and Indonesian and Spanish women. Somewhat lower correlations (compared to those between Spanish and American women) were shown for the dimensions of Arousal and Dominance, yet the correlational estimates were comparable to those of American and Spanish women.

**FIGURE 9:**
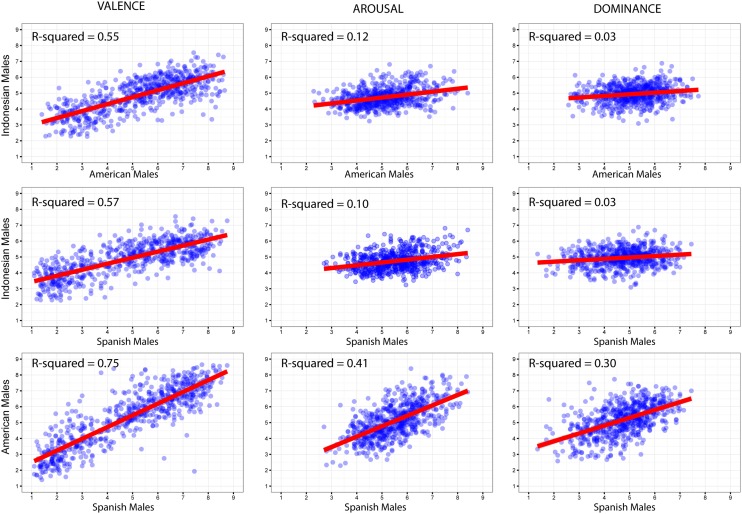
**Cross-cultural regression analyses on men’s ratings**.

However, when we look at **Figure 9**, the cross-cultural correlational results were surprisingly lower in men, ranging from *r* = 0.17, *R*^2^ = 0.03 to *r* = 0.75, *R*^2^ = 0.57 (all *p*s < 0.001). Consistent with the patterns of women, Indonesian men’s ratings showed their highest correlations with the ratings of American and Spanish men on the Valence dimension. However, the correlations yielded low estimates of effect size for Arousal and Dominance dimensions, compared to those of American and Spanish men. The estimates for cross-cultural correlations of Indonesian men were also lower than what Redondo et al. (2007) and Soares et al. (2012) found in their studies on men’s ratings across cultures.

The observed larger cultural differences in men than in women might be due to the lower range of Arousal and Dominance values given by Indonesian men (**Figure 9**). This finding suggests that these men tended to avoid extreme Arousal and Dominance values. In contrast, men from the United States and Spain tended to express their ratings across the whole scale. In other words, these patterns seemingly reflect a higher emotional reactivity, specifically in the expression of arousal and dominance aspects of emotion, in American and Spanish men than in Indonesian men.

As Montefinese et al. (2014) has suggested, differences in arousal might indeed be related to the different levels of emotional response and control, reflected in this study in the Dominance ratings. Such differences might be salient between individuals who come from individualistic and collectivistic cultures: in the latter group, gender roles are more traditional and strongly adhered to in terms of emotional response and control in daily interactions. This is apparently manifested strongly among male speakers. Therefore, the cultural differences between Indonesian and Spanish or American men might reflect the cross-cultural findings on emotional response and control collected through self-report methods in individualistic and collectivistic cultures (e.g., Levenson et al., 1992). The patterns we observed in the ratings of Arousal and Dominance in Indonesian men are highly relevant for emotion studies, because they point to possible cultural and gender differences in our attempts to understand fundamental aspects of emotion processes. A recent cross-cultural study on facial expression also showed that cultural rules that endorse individuals to openly express their emotion might be attuned to the long-history migration in a cultural society (Rychlowska et al., 2015). Such evidence shows that cultural variation in emotion expression is now considered as an important aspect in global communication.

The usefulness of the dimensional approach to emotion has stimulated research interest in the interaction of emotion and cognition. However, published affective word ratings have been mostly collected for members of individualistic cultures. A study that compared the processing of words’ affective dimensions in Chinese (Skrandies and Chiu, 2003) and German speakers (Skrandies, 1998) reported that the two groups of speakers elicited comparable event-related potential components and latencies, associated with the different stages of stimulus processing. Yet, whether the comparable results in Skrandies (1998) and Skrandies and Chiu (2003) studies were driven by the same set of word stimuli or not was not reported. As such, it is hard to assess the extent to which the emotional content of Chinese and German words is in fact associated with comparable neurophysiological activity across members of the two cultures.

Another appealing example is based on evidence that the contents of autobiographical memory are affected by sociocultural learning in terms of the relation of one’s self to others (Ross and Wang, 2010). As a type of declarative memory, autobiographical memory also contributes to the formation of semantic memory, which stores all abstract semantic representations of knowledge, including emotional stimulus aspects (Binder and Desai, 2011). In addition, ample evidence has shown that the activity of emotion-related neural structures such as hippocampus and amygdala is associated with the declarative memory processes during encoding-recognition (e.g., Ritchey et al., 2013). Yet, to what extent neurophysiological activity during the emotion recognition of encountered verbal stimuli is similar across cultures, is still unknown. One possibility is that the affective aspects of stimuli activate similar emotion-perceptual-related neurophysiological processes that are not correlated with cultural expectations of emotional expression in ratings (see also Fiske, 2000).

To the best of our knowledge, these problems have not received much attention in current emotion-laden word processing studies. Do affective ratings of written words reflect emotion-specific processes in (a) bodily and neurophysiological states; (b) socio-psychological states dependent on culture-specific circumstances and gender roles; and/or (c) cultural prescriptions in emotional display rules in general? At the same time, differences in emotional responses collected by means of self-report methods have been reported in many previous social psychological studies. It is therefore worthwhile to test whether such cultural differences on words’ ratings that might be contingent on gender, implicate differences in the conscious, cultural display rules of emotion or in the deeply rooted physiological or neural resonance of emotion. A well-controlled study involving speakers from cultures that are distant in terms of adherence to traditional gender roles can address this topic.

## Conclusion

In the present study, we introduced the first set of affective and psycholinguistic norms for Indonesian words (CEFI) based on subjective ratings by Indonesian speakers. The set shows universal patterns with respect to the relations of Valence and Arousal, as well as Valence and Dominance, thus replicating findings in many European languages and American English. The relation of Arousal and Dominance also showed a similar pattern in English words. Moreover, an increase in Predictability level of affective meaning was associated with an increase in Valence and Dominance, and a decrease in Arousal levels. These findings suggest that the affective properties of words as proposed by dimensional models and appraisal theories of emotion are rather universal properties of word meanings. We further found that the interrelationship of affective and non-affective dimensions is also similar to that in other published European and American English word datasets. In line with these findings, we expect our rating study to be useful for stimulus selection purposes in future studies on Indonesian word processing. Finally, we found interesting variations in affective word properties across cultures for male speakers in Indonesia, United States, and Spain. We therefore recommend that future studies on emotion and language processes are directed toward understanding the roles of culture and gender.

## Ethics Statement

This study was approved by the Ethics Committee of the Faculty of Social Sciences (ECSW) of Radboud University Nijmegen (ECG2012-3008-043: Verwerving van een vreemde taal) on August 30th, 2012. The study was conducted using a web-survey procedure. By way of introduction, participants were explicitly informed about the goals of the survey, the name of the researchers, and e-mail contacts. The participants were also informed about the filling and rating instructions, the estimated time to complete the survey, and the confidentiality of personal data. The participants were explicitly informed that they were free to participate and to quit in the middle of completing a questionnaire. Finally, if they liked to participate in the survey, they were asked to click on the button “submit”. We did not involve minors, persons with disabilities or endangered animal species.

## Author Contributions

AS designed, conducted the study, analyzed the data, and wrote the manuscript. PG designed and conducted the study and provided the data acquisition software. TD designed and conducted the study and wrote the manuscript. All co-authors approved the manuscript.

## Conflict of Interest Statement

The authors declare that the research was conducted in the absence of any commercial or financial relationships that could be construed as a potential conflict of interest.
