# Supplementary material for: Affective Meaning, Concreteness, and Subjective Frequency Norms for Indonesian Words
Source: Front Psychol. 2016 Dec 6;7:1907. doi: 10.3389/fpsyg.2016.01907 (PMC5138238; doi:10.3389/fpsyg.2016.01907)
Supplement: Supplementary file 2 [file Data_Sheet_2.PDF]

VALENSI

Mohon terlebih dahulu anda baca hal-hal berikut ini sebelum memulai. Pada setiap kata, anda akan melihat sebuah skala di sisi kanan kata tersebut. Skala ini dimulai dari perasaan sangat negatif (kiri) hingga sangat positif (kanan). Anda diminta untuk membaca setiap kata lalu memberikan penilaian tentang bagaimana perasaan anda saat membaca kata tersebut dengan mengklik salah satu lingkaran yang sesuai pada skala yang tersedia. Di lingkaran paling kiri, anda merasa tidak bahagia, kesal, tidak puas, melankolis, putus asa, atau bosan. Di lingkaran paling kanan, anda merasa bahagia, gembira, puas, senang, atau penuh harapan.

Sejauh mana anda memiliki perasaan positif atau negatif untuk setiap kata berikut ini?

sangat negatif							sangat positif	
1	2	3	4	5	6	7	8	9

GAIRAH

Mohon terlebih dahulu anda baca hal-hal berikut ini sebelum memulai. Pada setiap kata, anda akan melihat sebuah skala di sisi kanan kata tersebut. Skala ini dimulai dari perasaan tenang (kiri) hingga tergugah (kanan). Anda diminta untuk membaca setiap kata lalu memberikan penilaian tentang bagaimana perasaan anda saat membaca kata tersebut dengan mengklik salah satu lingkaran yang sesuai pada skala yang tersedia. Di lingkaran paling kiri, anda merasa betul-betul rileks, tenang, lamban, kuyu, mengantuk, atau tidak tergerak. Di lingkaran paling kanan, anda merasa terstimulasi, bersemangat, meluap-luap, tidak tenang, bergairah, atau aktif.

Sejauh mana anda merasa tenang atau tergugah untuk setiap kata berikut ini?

tenang								aktif
1	2	3	4	5	6	7	8	9

KEKONKRETAN

Mohon terlebih dahulu anda baca hal-hal berikut ini sebelum memulai. Pada setiap kata, anda akan melihat sebuah skala di sisi kanan kata tersebut. Skala ini dimulai dari konkret (kiri) hingga abstrak (kanan). Anda diminta untuk membaca setiap kata lalu memberikan penilaian anda tentang kata tersebut dengan mengklik salah satu lingkaran yang sesuai pada skala yang tersedia. Di lingkaran paling kiri, anda berpendapat bahwa kata tersebut merujuk pada sesuatu yang bisa anda sentuh, lihat, atau anda rasakan secara fisik. Di lingkaran paling kanan, anda berpendapat bahwa kata tersebut merujuk pada sesuatu yang tidak dapat disentuh, lihat, atau alami secara fisik.

Sejauh mana setiap kata berikut ini konkret atau abstrak?

konkret								abstrak
1	2	3	4	5	6	7	8	9

FREKUENSI SUBYEKTIF

Mohon terlebih dahulu anda baca hal-hal berikut ini sebelum memulai. Pada setiap kata, anda akan melihat sebuah skala di sisi kanan kata tersebut. Skala ini dimulai dari sangat jarang (kiri) hingga sangat sering (kanan). Anda diminta untuk membaca setiap kata lalu memberikan penilaian anda tentang kata tersebut dengan mengklik salah satu lingkaran yang sesuai pada skala yang tersedia. Di lingkaran paling kiri, anda berpendapat bahwa kata tersebut sangat jarang anda temukan dalam kehidupan sehari-hari. Di lingkaran paling kanan, anda berpendapat bahwa kata tersebut sangat sering anda temukan dalam kehidupan sehari-hari.

Sejauh mana setiap kata berikut ini anda temukan dalam kehidupan sehari-hari?

sangat jarang								sangat sering
1	2	3	4	5	6	7	8	9

KEKUASAAN

Mohon terlebih dahulu anda baca hal-hal berikut ini sebelum memulai. Pada setiap kata, anda akan melihat sebuah skala di sisi kanan kata tersebut. Skala ini dimulai dari perasaan lemah dan dikendalikan (kiri) hingga kuat dan mengendalikan (kanan). Anda diminta untuk membaca setiap kata lalu memberikan penilaian tentang bagaimana perasaan anda saat membaca kata tersebut dengan mengklik salah satu lingkaran yang sesuai pada skala yang tersedia. Di lingkaran paling kiri, anda merasa lemah dan dikendalikan, terpengaruh, atau tunduk. Di lingkaran paling kanan, anda merasa kuat dan betul-betul punya kendali, dominan, atau mandiri.

Sejauh mana anda merasa lemah dan dikendalikan atau kuat dan mengendalikan untuk setiap kata berikut ini?

lemah dan dikendalikan								kuat dan mengendalikan
1	2	3	4	5	6	7	8	9

KETERDUGAAN

Mohon terlebih dahulu anda baca hal-hal berikut ini sebelum memulai. Pada setiap kata, anda akan melihat sebuah skala di sisi kanan kata tersebut. Skala ini dimulai dari perasaan sangat tak terduga (kiri) hingga sangat terduga (kanan). Anda diminta untuk membaca setiap kata lalu memberikan penilaian tentang bagaimana perasaan anda saat membaca kata tersebut dengan mengklik salah satu lingkaran yang sesuai pada skala yang tersedia. Di lingkaran paling kiri, anda merasakan suatu perasaan yang sangat tak terduga dan mendorong anda untuk bereaksi secara spontan. Di lingkaran paling kanan, anda merasakan suatu perasaan yang sangat terduga dan tidak sedikit pun memberikan anda dorongan untuk bereaksi.

Sejauh mana anda merasakan perasaan yang tak terduga atau terduga untuk setiap kata berikut ini?

sangat tak terduga								sangat terduga
1	2	3	4	5	6	7	8	9
