# Supplementary material for: Affective Meaning, Concreteness, and Subjective Frequency Norms for Indonesian Words
Source: Front Psychol. 2016 Dec 6;7:1907. doi: 10.3389/fpsyg.2016.01907 (PMC5138238; doi:10.3389/fpsyg.2016.01907)
Supplement: Supplementary file 3 [file Data_Sheet_3.PDF]

VALENCE

Please first read the following items before starting. In every word, you will see a scale on the right side of the word. This scale starts from feeling very negative (left) to very positive (right). You are required to read each word and give an assessment of how you feel when you read the word by clicking one of the circles corresponding to the scale provided. On the far left circle, you feel unhappy, upset, unhappy, melancholy, despair, or bored. On the far right circle, you feel happy, excited, pleased, happy or hopeful.

To what extent do you have positive or negative feelings for each of the following words?

very negative								very positive
1	2	3	4	5	6	7	8	9

AROUSAL

Please first read the following items before starting. In every word, you will see a scale on the right side of the word. This scale starts from a feeling of calm (left) to intrigued (right). You are required to read each word and give an assessment of how you feel when you read the word by clicking one of the circles corresponding to the scale provided. On the far left circle, you feel really relaxed, calm, slow, dull, sleepy, or unmoved. On the far right circle, you feel stimulated, excited, overflowing, restless, aroused or active.

To what extent do you feel calm or aroused for each of the following words?

calm								active
1	2	3	4	5	6	7	8	9

CONCRETENESS

Please first read the following items before starting. In every word, you will see a scale on the right side of the word. This scale starts from concrete (left) to the abstract (right). You are required to read each word and then give your judgment about it by clicking one of the circles corresponding to the scale provided. On the far left circle, you found the word refers to something that you can touch, see, or you feel physically. On the far right circle, you found the word refers to something that can not be touched, seen, or experienced physically.

To what extent do you think that each of the following words is concrete or abstract?

concrete								abstract
1	2	3	4	5	6	7	8	9

SUBJECTIVE FREQUENCY

Please first read the following items before starting. In every word, you will see a scale on the right side of the word. This scale starts from a very rare (left) to very often (right). You are required to read each word and then give your judgment about it by clicking one of the circles corresponding to the scale provided. On the far left circle, you found the word very rarely be found in everyday life. On the far right circle, you found the word very often be found in everyday life.

To what extent do you encounter each of the following words in daily life?

very rarely								very frequently
1	2	3	4	5	6	7	8	9

DOMINANCE

Please first read the following items before starting. In every word, you will see a scale on the right side of the word. This scale starts from feeling weak and controlled

(left) up to a powerful and controlling (right). You are required to read each word and give an assessment of how you feel when you read the word by clicking one of the circles corresponding to the scale provided. On the far left circle, you feel weak and controlled, influenced, or dependent. On the far right circle, you feel strong and fully in control, dominant, or independent.

To what extent do you feel weak and controlled or strong and controlling for each of the following words?

weak and controlled								strong and in control
1	2	3	4	5	6	7	8	9

PREDICTABILITY

Please first read the following items before starting. In every word, you will see a scale on the right side of the word. This scale starts from highly unpredictable (left) to highly predictable (right). You are required to read each word and give an assessment of how you feel when you read the word by clicking one of the circles corresponding to the scale provided. On the far left circle, you experience a sense of a highly unpredictable feeling that encourages you to react spontaneously. On the far right circle, you experience a sense of a highly predictable feeling that does not the slightest give you the urge to react.

To what extent do you feel a sense of unpredictability or a sense of predictability for any of the following words?

highly unpredictable							highly predictable	
1	2	3	4	5	6	7	8	9
